# Predictors of fear of diabetes progression: A multi-center cross-sectional study for patients self-management and healthcare professions education

**DOI:** 10.3389/fpubh.2022.910145

**Published:** 2022-12-19

**Authors:** Yanhao Wang, Qiuhua Yu, Zihuan Zeng, Ruizhu Yuan, Ruiding Wang, Jianli Chen, Hengyu Zhou, Jiao Tang

**Affiliations:** ^1^Key Laboratory of Shaanxi Province for Craniofacial Precision Medicine Research, College of Stomatology, Xi'an Jiaotong University, Xi'an, Shaanxi, China; ^2^Chongqing Key Laboratory for Oral Diseases and Biomedical Sciences, College of Stomatology, Chongqing Medical University, Chongqing, China; ^3^Department of Orthodontics, School of Stomatology, Xi'an Jiaotong University, Xi'an, Shaanxi, China; ^4^Department of Breast Surgery, Peking Union Medical College Hospital (Xidan Campus), Beijing, China; ^5^State Key Laboratory of Trauma, Burn and Combined Injury, Institute of Burn Research, Southwest Hospital, Third Military Medical University (Army Medical University), Chongqing, China; ^6^Department of Plastic Surgery, State Key Laboratory of Trauma, Burns and Combined Injury, Southwest Hospital, Third Military Medical University (Army Medical University), Chongqing, China; ^7^School of Nursing, Chongqing Medical University, Chongqing, China; ^8^Department of Critical Care Medicine, Shanghai Jiao Tong University Affiliated Sixth People's Hospital, Shanghai, China; ^9^Guangzhou Women and Children Medical Center, Guangzhou Medical University Affiliated Women and Children Medical Center, Guangzhou, China; ^10^Department of Nursing, The First Affiliated Hospital of Chongqing Medical University, Chongqing, China

**Keywords:** predictors, fear of progression, type 2 diabetes mellitus (T2DM), multi-center, cross-sectional study, healthcare professions education, self-management

## Abstract

**Objective:**

Excessive fear of progression can affect the mental health, social function, and wellbeing of patients with chronic diseases. This study investigated the fear of progression (FoP) and the socio-demographic and clinical predictors among patients with type 2 diabetes mellitus (T2DM).

**Method:**

The present study is a multi-center cross-sectional study. Inpatients with T2DM were recruited by a multi-stage convenience sampling method from the department of endocrinology in 5 tertiary hospitals in Southwest China. 459 T2DM patients were consecutively enrolled. Socio-demographic, clinical data, and answers to the fear of progression questionnaire (FoP-Q) were collected.

**Results:**

385 patients with complete data were eligible. The average score of FoP-Q-SF was 26.84 and 23.1% of patients reached the dysfunctional fear of progression criterion. The greatest fears were worrying about “disease progression,” “the adverse reactions of medication,” and “relying on strangers for activities of daily living.” Health education (*P* < 0.001), age (*P* = 0.002), hypoglycemia history (*P* = 0.006), employment status (*P* = 0.025) and duration since being diagnosed with type 2 diabetes mellitus (*P* = 0.032) were the related factors of fear of progression.

**Conclusion:**

Early assessment of the fear of progression was imperative to identify dysfunctional fear of progression in patients with type 2 diabetes mellitus. Meanwhile, the meaning of these predictors for strengthening healthcare professions education and patients self-management might help healthcare givers timely perform related interventions and help patients reduce their fear of progression thus actively cooperate with T2DM treatments.

## Introduction

Diabetes mellitus is a global epidemic with ~463 million adults worldwide suffering from the disease (1). China is the country with the largest number of diabetic people in the world, and the prevalence of diabetes in China in 2019 was 10.9% (1). Type 2 diabetes mellitus (T2DM), as the most common type of diabetes, the incidence will increase with the aging population (1, 2).

T2DM patients face various threats. First is the complexity of the diabetes therapeutic schedule, which combines diet, exercise, medication, blood sugar testing, oral care, and foot care (1, 3). Moreover, the age-standardized disability-adjusted life-years of diabetes was lower than expected in China in 2017, but all-cause mortality in diabetic patients was still significantly higher than in people without diabetes (4, 5). T2DM patients have a high risk of multiple crucial organ damage because of long-term hyperglycemia, which increases hospitalization rates and the economic burden on patients (2, 6, 7). These problems are not imaginary because of patients' mental anxiety, but real in daily life. Patients are facing real threats, and the resulting fear is neither inappropriate nor irrational. The above threats often lead to patients with T2DM experiencing negative emotional outcomes.

Fear of progression (FoP) refers to the patients' fear of biological or psychological consequences due to the development or recurrence of their diseases, which is a fully conscious, non-neurophobic response (8–10). The level of FoP often ranges between functional and dysfunctional ends, when it elevates to a dysfunctional degree (e.g., affecting treatment or life), it is necessary to take measures. It grows recognition that FoP is one of the significant emotional difficulties facing patients with chronic progressive diseases (8), and it is also a disease experience that threatens the life of individuals (10, 11). Moreover, high FoP will affect the patient's behavioral dysfunction, wellbeing, mental health, and health-related quality of life (9, 10, 12, 13).

Herschbach et al. (14) formally proposed the Fear of Progression Questionnaire (FoP-Q) to determine FoP in patients with cancer, diabetes mellitus, and rheumatic diseases. Researches have explored the FoP and its related factors in patients with chronic diseases, especially in malignant tumors. Previous studies found that cancer patients and their partners had higher FoP than other chronic progressive diseases (e.g., diabetes mellitus) (8, 11, 15). Furthermore, the level of FoP was consistently related to their age, education status, new physical symptoms, employment status, and income (16–20). Kalra et al. (21) defined that disease progression of T2DM includes not only a gradual deterioration of cellular function, but also an increase in the dosage, frequency, and the amount of drugs used, and the occurrence or worsening of complications. Herschbach et al. suggested that diabetic patients shared specific fears about the future course of their illness (14), and the study of Dankert et al. indicated that fear of an increasing disease progression (fear of progression) was among the primary psychological distresses in patients with diabetes mellitus (8). Moreover, concrete consequences of dysfunctional fear of progression in diabetes mellitus may include affecting the mental health and quality of life of patients, and the emotional reactions of the partner of patients (8, 11).

In short, FoP is vital for the physical and mental health of patients with diabetes mellitus. However, it is poorly known about FoP and its related factors among patients with T2DM in mainland China. Therefore, our study intended to investigate FoP and explore its related factors. The results of this study may enhance understanding of the status of FoP in patients with T2DM and help healthcare givers develop more effective interventions and strategies to avoid the adverse effects of FoP.

## Methods

### Participants and procedures

The present cross-sectional survey was held in 2019. The sample size was determined through power analysis and calculated using the G^*^Power program (22). Linear multiple regression in the G^*^Power program was conducted using a random model. When using a two-tailed test, considering an effect size of 0.10, 13 independent variables related to FoP based on the relevant published work (16, 19, 20), significance level (*p*) of 0.05, and 95% power, at least 287 participants were required.

All the inpatients with T2DM were recruited by multi-stage convenience sampling method from the department of endocrinology in five tertiary hospitals in Southwest China. The inclusion criteria were listed below:

(1) Diagnosis of type 2 diabetes;
(2) Age ≥ 18 years;
(3) Education level of primary school or above;
(4) Informed consent.

And the exclusion criteria were following:

(1) T2DM with pregnancy or malignant tumors;
(2) History of mental illness and family history;
(3) Other serious medical conditions make it difficult to talk and fill out questionnaires.

Finally, 426 T2DM patients were enrolled from five tertiary hospitals in Southwest China, and 385 patients with complete data were eligible for data analysis.

### Data collection

#### Socio-demographic and clinical data

Socio-demographic characteristics were collected including age, gender, marital status, education attainment, and employment status. Clinical characteristics were collected from patients' medical records.

#### Fear of progression questionnaire-short form

FoP-Q-SF, developed by Mehnert et al. (23), is a 12-item self-reporting questionnaire. Each item is rated on a 5-point Likert scale ranging from 1 point (never) to 5 points (very often). The overall score is calculated as the sum of all item scores. Patients will suffer from psychological dysfunction when the overall score is ≥34 points in the FoP-Q-SF (10). Moreover, FoP-Q-SF has been translated into multiple languages, and it is an appropriate and valuable tool for measuring FoP (14, 24, 25). The psychometric properties of the Chinese version of FoP-Q-SF among patients with T2DM have been verified by Zeng et al. (25). The Cronbach's α coefficient, split-half reliability, and test-retest reliability were 0.876, 0.828, and 0.837, separately (25). FoP-Q-SF had satisfactory psychometric properties in Chinese patients with T2DM.

### Data analysis

Data were analyzed using the Statistical Package for the Social Sciences (SPSS) version 25.0 (IBM Corporation, Armonk, NY, USA). Descriptive statistics were used to calculate the sample characteristics and FoP-Q-SF score. For univariate analysis, the independent-sample *t*-test and one-way analysis of variance (ANOVA) were conducted to contrast the differences in FoP (continuous variables in this study showing a normal distribution). Subsequently, the variables with *P* < 0.05 in the univariate analysis were included in the multiple linear regression model to determine factors independently associated with FoP. Notably, the following assumptions were tested before multiple linear regression analysis: (1) drawing scatter plots to test the linearity of the model; (2) A histogram to test the normally distributed standardized residuals; (3) a Durbin-Watson value of about two to verify no autocorrelation in the residuals; (4) variance inflation factor (VIF) < 10 to verify no multicollinearity between independent variables. If all assumptions were satisfied, multiple linear regression analysis would be used.

### Ethical considerations

All the study procedures were reviewed and approved by the Ethics Committee of the target University. Informed consent has been obtained from the patients or their guardians before data collection. The research conforms to the provisions of the Declaration of Helsinki in 1995 (as revised in Edinburgh 2000).

## Results

### Socio-demographic and clinical characteristics

As is shown in Table 1, a total of 385 patients completed this study. The mean age was 57.65 (SD = 15.15). Many patients (More than 60%) are unemployed, including more than 70% of retirees. The mean duration since being diagnosed of the patients was 9.93 years (SD = 7.54), and the mean body mass index was 24.06 (SD = 3.22). Most of the patients (90.6%) had poor glycemic control (HbA1c ≥ 7%) (Table 1).

**Table 1:** Socio-demographic and clinical characteristics of the type 2 diabetic patients (*n* = 385).

**Characteristic**	***N***	**%**
**AGE (YEARS)**		
Mean (SD) = 57.65 (15.15)		
< 45.0	74	19.2
45.0–59.9	123	32.0
≥60	188	48.8
**Gender**		
Male	189	49.1
Female	196	50.9
**Marital status**		
Married	334	86.8
Unmarried	51	13.2
**Educational attainment**		
Primary school	105	27.3
High school	184	47.8
College or above	96	24.9
**Employment status**		
Employed	137	35.6
Unemployed	248	64.4
Retired	177	71.4
Others	71	28.6
**Monthly household income (per person per month)**		
< ¥3,000	140	36.4
¥3,000–5,999	171	44.4
≥¥6,000	74	19.2
**Number of hospital admittances because of T2DM at enrolment**		
First time	88	22.9
Second time or more	297	77.1
**Receiving health education on diabetes**		
Yes	239	62.1
No	146	37.9
**Hypoglycemia history**		
Yes	222	57.7
No	163	42.3
**DURATION SINCE DIAGNOSIS (YEARS)**		
Mean (SD) = 9.93 (7.54)		
< 5	122	31.7
5–9.9	103	26.7
≥10	160	41.6
**Diabetes treatment type**		
Oral medication	118	30.6
Insulin	66	17.1
Oral medication + insulin	201	52.2
**BODY MASS INDEX (KG/M** ^**2**^ **)**		
Mean (SD) = 24.06 (3.22)		
< 18.5	12	3.1
18.5–23.9	176	45.7
24–27.9	157	40.8
≥28	40	10.4
**HbA1c (%) (*****N*** **=** **363)**		
Mean (SD) = 10.32 (2.02)		
< 7	14	3.6
≥7	349	90.6

HbA1c, glycated hemoglobin.

### Fear of progression

Table 2 shows FoP among patients with T2DM. The mean score of FoP was 26.84 (SD = 8.94). Eighty-nine patients (23.1%) had the score of FoP-Q-SF ≥ 34 points. The three items, named “Being afraid of disease progression,” “Worrying that the adverse reactions of medication,” and “Relying on strangers for activities of daily living,” with the highest score were 2.66 (SD = 1.20), 2.44 (SD = 1.25), and 2.36 (SD = 1.22) (Table 2).

**Table 2:** Fear of progression in the type 2 diabetic patients (*n* = 385).

**Fear of progression**	**Mean (SD)**
1. Being afraid of disease progression	2.66 (1.20)
10. Worrying that medication could damage the body	2.44 (1.25)
7. Being afraid of relying on strangers for activities of daily living	2.36 (1.22)
2. Being nervous prior to doctor's appointment or periodic examinations	2.10 (1.09)
9. Being afraid of severe medical treatments in the course of the illness	2.27 (1.20)
3. Being afraid of pain	2.25 (1.22)
6. Being afraid by the possibility that the children could contract the disease	2.21 (1.22)
5. Having physical symptoms (e.g., rapid heartbeat, stomach ache)	2.20 (1.14)
11. Worrying what will become of family if something happens to me	2.18 (1.21)
12. Being afraid of not being able to work anymore	2.08 (1.28)
8. Being afraid of no longer being able to pursue hobbies	2.06 (1.14)
4. Being afraid of becoming less productive at work	2.03 (1.22)
Total score	26.84 (8.94)

### Predictors of fear of progression

Univariate analysis identified a range of factors that were significantly associated with FoP: patients who aged <60, who were employed, and who had higher educational attainment reported higher FoP (*P* < 0.001). Patients hospitalized twice or more due to T2DM showed a higher level of FoP (*P* = 0.009). Patients had a higher level of FoP if they had hypoglycemia history, higher HbA1c, shorter time since being diagnosed with T2DM, and did not receive health education (*P* < 0.001) (Table 3).

**Table 3:** Differences in fear of progression among various socio-demographic and clinical sub-groups (*n* = 385).

**Variable**	***n***	**Fear of progression**		
		**Mean (SD)**	***t***/***F***	***P***
**Age (years)**			40.679	0.000
< 45.0	74	32.22 (7.11)		
45.0–59.9	123	27.37 (8.51)		
≥60	188	24.37 (8.91)		
**Employment status**			4.944	0.000
Employed	137	29.78 (7.97)		
Unemployed	248	25.21 (9.05)		
**Educational attainment**			6.088	0.002
Primary school	105	36.41 (11.06)		
High school	184	37.55 (10.34)		
College or above	96	41.21 (9.93)		
**Number of hospital admittances**			2.643	0.009
**because of T2D at enrolment**		
First time	88	29.03 (6.01)		
Second time or more	297	26.19 (9.55)		
**Receiving health education on**			−8.024	0.000
**diabetes**		
Yes	239	29.54 (7.94)		
No	146	22.42 (8.75)		
**Hypoglycemia history**			−5.050	0.000
Yes	222	28.76 (8.52)		
No	163	24.22 (8.86)		
**Duration since diagnosis (years)**			16.132	0.000
< 5	122	29.94 (9.537)		
5–9.9	103	27.39 (7.758)		
≥10	160	24.12 (8.377)		
**HbA1c (%) (*****n*** **=** **363)**			−4.227	0.000
< 7	14	17.07 (5.413)		
≥7	349	27.13 (8.829)		

HbA1c, glycated hemoglobin.

Then, the above variables with *P* < 0.05 were included in the multiple linear regression model. And the best-fit multiple linear regression model included five variables (Table 4). Receiving health education (β = 0.285, *P* < 0.01), age (β = −0.186, *P* = 0.002), hypoglycemia history (β = −0.136, *P* = 0.006), employment status (β = −2.126, *P* = 0.025) and duration since being diagnosed with T2DM (β = −0.107, *P* = 0.032) were the predictors of FoP. The variables co–explained the 26.8% variation of FoP.

**Table 4:** Predictors of fear of progression among type 2 diabetic patients (*n* = 385).

**Variable**	***B***	**SE-***B*****	**β**	***t***	***P***	**VIF**
Constant	25.275	4.583	–	5.514	0.000	–
Receiving health education on diabetes^†^	5.287	0.949	0.285	5.569	0.000	1.297
Age	−0.108	0.034	−0.186	−3.182	0.002	1.695
Hypoglycemia history^†^	−2.452	0.879	−0.136	−2.790	0.006	1.179
Employment status^†^	−2.126	0.947	−0.115	−2.246	0.025	1.285
Duration since diagnosis	−0.127	0.059	−0.107	−2.158	0.032	1.212

*B*, unstandardized coefficient beta; SE-*B*, standard error of *B*; β, standardized coefficient beta; VIF, variance inflation factor.

† Receiving health education on diabetes: 1 = Yes, 2 = No; Hypoglycemia history: 1 = Yes, 2 = No; Employment status: 1 = employed, 2 = Unemployed.

## Discussion

FoP is an appropriate response to the real threats of diagnosis, treatment, and course of illness, and nearly all patients have feelings ranging from very mild upset to severe worries. Nevertheless, excessive fear can induce significant adverse effects on glycemic control, behavioral dysfunction, wellbeing, and health-related quality of life in diabetic patients (9, 10, 12, 13, 26). Patients with T2DM rely on a complexity of diabetes therapeutic schedule (e.g., a combination of multiple drugs and/or insulin therapy) to achieve glycemic control (1, 3). With the development of T2DM, original symptoms become aggravated and new symptoms or complications will appear to reduce the quality of life (e.g., high risk of disability). These reasons may lead to patients' high FoP. Therefore, it is essential to understand and control FoP in patients with T2DM.

We can use questionnaire scores to initially assess the degree of fear in patients. In this study, the score of FoP-Q-SF was lower than a Chinese study among 636 cancer patients (20), and a German study among partners of chronically ill patients (11). A possible explanation is that T2DM is a common disease, and there are currently mature treatment plans. Although it cannot be completely cured, patients can delay the progression of the disease and control its recurrence through various methods, which significantly reduces the psychological pressure of T2DM patients.

Moreover, 23.1% of patients in our study developed psychological dysfunction due to FoP. The findings of the present study suggested that FoP among patients with T2DM, especially those with psychological dysfunction, need paid more attention to and timely identified using FoP-Q-SF by health caregivers to distinguish with anxiety disorder. Previous studies also found that health caregivers might use protective motivation theory and cognitive behavioral psychotherapy to reduce patients' FoP (11, 27).

In the present study, the three items with the highest score were “Being afraid of disease progression,” “Being worried about side effects of medication,” and “Relying on strangers for activities of daily living.” However, the findings in our study were different from brain cancer patients, and they worried about “what will become of family if something happens to me” and “severe medical treatments in the course of the illness” (28). This difference may be related to the different psychology of patients caused by different severity and prognosis of the disease. Patients with diabetes have better prognosis and less serious life-threatening conditions, so the patients are more concerned about changes in quality of life, while cancer patients are more concerned about survival rates and the impact on their families. Therefore, different psychological problems should be addressed for patients with different chronic diseases. Furthermore, a significant discrepancy between our research and a previous study from Thailand is that being afraid of relying on strangers for activities of daily living is the most infrequent fear in the previous study, even though there was a common phenomenon that three or four generations under one roof (29), which may be related to our traditional culture.

People from different backgrounds have different levels of FoP, probably due to different levels of awareness of the disease. Therefore, when we make a control plan, it is essential to be concerned about their sociodemographic elements. Our study found that health education is crucial to avoiding high FoP. Health education on diabetes is recognized as an essential measure to improve knowledge and awareness of diabetes and further reduce the risk of developing FoP (30–32). However, more than 35.0% of patients in our study had not received health education on diabetes. Winkley et al. (33) found that newly diagnosed patients with T2DM showed low attendance at structured education, which might increase their fear of the unknown disease. This result could also be ascribed to the fact that the number of qualified diabetes educators was very limited in China. Considering the massive population of individuals with diabetes in mainland China, cultivating more high-quality certified diabetes educators is warranted. Moreover, limited health resources can be used maximum by health caregivers with new technology (e.g., online health education based on mobile phones or computers) (34, 35).

The other clinical related factor of FoP is hypoglycemia history. Hypoglycemia is a common complication of diabetes treatment, and it often accompanies unpleasant experiences and negative consequences (36). Further, severe hypoglycemia is associated with the rapid progression of diabetes (37), even cause serious acute and chronic complications. Close monitoring of blood glucose might be used to avoid FoP due to hypoglycemia history (38).

Our study showed that age and employment status were related to FoP. Patients who were younger and employed had a higher level of FoP. At present, most employed patients with T2DM are middle-aged (39). Patients often thought that their life expectancy might be longer, and the negative impact of T2DM would be more far-reaching (40), but Seuring et al. (41) found that T2DM had limited their labor productivity, particularly in developing countries. In China, patients with T2DM, employed in recent decades, were born in the period of implementation of the one-child policy and faced unprecedented financial pressure (e.g., children's tuition and parents' pensions) (42). The above reasons might result in their higher level of FoP.

This study investigated the source and extent of FoP among T2DM patients, and further clarified FoP related factors. The present study's findings may be useful for targeting and tailoring FoP interventions of specific population groups.

Despite the supportive finding of the study, there were some limitations need to be acknowledged. Compared with outpatients, hospitalized T2DM may be a more serious condition (e.g., higher HbA1C). Thus, we only enrolled inpatients with T2DM from the department of endocrinology in five tertiary hospitals in Southwest China, the ability to generalize the study results may be limited. Furthermore, the *R*^2^ value of the multiple linear regression model was relatively small, and further studies were warranted to identify other predictors of FoP.

## Conclusion

As a common chronic disease, T2DM poses great danger to human health. With its incidence increasing yearly, many prevention and treatment measures and educational activities have been carried out. More and more studies have focused on the negative psychological effects of the disease, including fear of all aspects of the disease (commonly referred to as fear of progression, FoP), in addition to its serious complications. Our study based on socio-demographic and clinical data, FoP-Q-SF completion, and data analysis of 385 patients. Finally, 23.1% of patients with T2DM reported high FoP. FoP among patients with T2DM varied from different socio-demographic and clinical characteristics, especially health education, hypoglycemia history, employment status, age and duration since being diagnosed with T2DM. Interventions should be based on patients varying socio-demographic and clinical profiles and tailored to the circumstances of the individual patients. The findings of this study may provide new insights on how to care for dysfunctional FoP patients with T2DM. Future research may continuously focus on the FoP of patients and their partners with T2DM, and for the predictors explore more effective interventions to decrease high FoP.

## Data availability statement

The original contributions presented in the study are included in the article/supplementary material, further inquiries can be directed to the corresponding authors.

## Ethics statement

The studies involving human participants were reviewed and approved by Ethics Committee of Chongqing Medical University. The patients/participants provided their written informed consent to participate in this study.

## Author contributions

YW and JT contributed to the conception and design of the data analysis and analyzed the data. YW and QY searched literature and prepared the manuscript. ZZ, RY, RW, and JC performed the investigation and collected the data. ZZ collated and supervised the data and other materials. YW, JT, and HZ prepared the revised manuscript. All authors have read and approved the manuscript.

## References

[1] International Diabetes Federation. IDF. Diabetes Atlas. 9th ed. (2019). Available online at: https://diabetesatlasorg/en (accessed December 6, 2021).

[2] Gregg EW Li Y Wang J Burrows NR Ali MK Rolka D. Changes in diabetes-related complications in the United States, 1990-2010. N Engl J Med. (2014) 370:1514–23. 10.1056/NEJMoa1310799 24738668

[3] Dobrica EC Gaman MA Cozma MA Bratu OG Pantea SA Diaconu CC. Polypharmacy in type 2 diabetes mellitus: insights from an internal medicine department. Medicina. (2019) 55:436. 10.3390/medicina55080436 31382651 PMC6723949

[4] Bragg F Holmes MV Iona A Guo Y Du H Chen Y. Association between diabetes and cause-specific mortality in rural and urban areas of China. JAMA. (2017) 317:280–9. 10.1001/jama.2016.19720 28114552 PMC6520233

[5] Zhou M Wang H Zeng X Yin P Zhu J Chen W. Mortality, morbidity, and risk factors in China and its provinces, 1990-2017: a systematic analysis for the Global Burden of Disease Study 2017. Lancet. (2019) 394:1145–58. 10.1016/S0140-6736(19)30427-1 31248666 PMC6891889

[6] Afroz A Alramadan MJ Hossain MN Romero L Alam K Magliano DJ. Cost-of-illness of type 2 diabetes mellitus in low and lower-middle income countries: a systematic review. BMC Health Serv Res. (2018) 18:972. 10.1186/s12913-018-3772-8 30558591 PMC6296053

[7] Hu WS Lin CL. Use of the progression of adapted Diabetes Complications Severity Index to predict acute coronary syndrome, ischemic stroke, and mortality in Asian patients with type 2 diabetes mellitus: a nationwide cohort investigation. Clin Cardiol. (2018) 41:1038–43. 10.1002/clc.22991 29896758 PMC6489707

[8] Dankert A Duran G Engst-Hastreiter U Keller M Waadt S Henrich G. Fear of progression in patients with cancer, diabetes mellitus and chronic arthritis. Rehabilitation. (2003) 42:155–63. 10.1055/s-2003-40094 12813652

[9] Herschbach P Dinkel A. Fear of progression. Recent results in cancer research. Psychooncology. (2014) 197:11–29. 10.1007/978-3-642-40187-9_2 24305766

[10] Hinz A Mehnert A Ernst J Herschbach P Schulte T. Fear of progression in patients 6 months after cancer rehabilitation-a- validation study of the fear of progression questionnaire FoP-Q-12. Support Care Cancer. (2015) 23:1579–87. 10.1007/s00520-014-2516-5 25412727

[11] Zimmermann T Herschbach P Wessarges M Heinrichs N. Fear of progression in partners of chronically ill patients. Behav Med. (2011) 37:95–104. 10.1080/08964289.2011.605399 21895427

[12] Donald M Dower J Coll JR Baker P Mukandi B Doi SA. Mental health issues decrease diabetes-specific quality of life independent of glycaemic control and complications: findings from Australia's living with diabetes cohort study. Health Qual Life Outcomes. (2013) 11:170. 10.1186/1477-7525-11-170 24131673 PMC3853250

[13] Grammes J Schafer M Benecke A Low U Klostermann AL Kubiak T. Fear of hypoglycemia in patients with type 2 diabetes: the role of interoceptive accuracy and prior episodes of hypoglycemia. J Psychosom Res. (2018) 105:58–63. 10.1016/j.jpsychores.2017.12.010 29332635

[14] Herschbach P Berg P Dankert A Duran G Engst-Hastreiter U Waadt S. Fear of progression in chronic diseases: psychometric properties of the Fear of Progression Questionnaire. J Psychosomat Res. (2005) 58:505–11. 10.1016/j.jpsychores.2005.02.007 16125517

[15] Hall DL Lennes IT Pirl WF Friedman ER Park ER. Fear of recurrence or progression as a link between somatic symptoms and perceived stress among cancer survivors. Support Care Cancer. (2017) 25:1401–7. 10.1007/s00520-016-3533-3 27966025 PMC5500975

[16] Crist JV Grunfeld EA. Factors reported to influence fear of recurrence in cancer patients: a systematic review. Psychooncology. (2013) 22:978–86. 10.1002/pon.3114 22674873

[17] Esser P Gotze H Mehnert-Theuerkauf A Knoop H Kuba K. Fear of progression and its role in the relationship of cancer-related fatigue with physical functioning and global quality of life - A register-based study among hematological cancer survivors. J Psychosomat Res. (2019) 127:109844. 10.1016/j.jpsychores.2019.109844 31707130

[18] Garcia S. The effects of education on anxiety levels in patients receiving chemotherapy for the first time: an integrative review. Clin J Oncol Nurs. (2014) 18:516–21. 10.1188/14.CJON.18-05AP 25164233

[19] Hefner J Csef EJ Kunzmann V. Fear of progression in outpatients with chronic myeloid leukemia on oral tyrosine kinase inhibitors. Oncol Nurs Forum. (2016) 43:190–7. 10.1188/16.ONF.190-197 26906130

[20] Yang Y Sun H Liu T Zhang J Wang H Liang W. Factors associated with fear of progression in chinese cancer patients: sociodemographic, clinical and psychological variables. J Psychosomat Res. (2018) 114:18–24. 10.1016/j.jpsychores.2018.09.003 30314574

[21] Kalra S Kamaruddin NA Visvanathan J Santani R. Defining disease progression and drug durability in type 2 diabetes mellitus. Eur Endocrinol. (2019) 15:67–9. 10.17925/EE.2019.15.2.67 31616495 PMC6785960

[22] Faul F Erdfelder E Lang AG Buchner A. G^*^Power 3: a flexible statistical power analysis program for the social, behavioral, and biomedical sciences. Behav Res Methods. (2007) 39:175–91. 10.3758/BF03193146 17695343

[23] Mehnert A Herschbach P Berg P Henrich G Koch U. Fear of progression in breast cancer patients–validation of the short form of the Fear of Progression Questionnaire (FoP-Q-SF). Zeitschrift Psychosomat Med Psychother. (2006) 52:274–88. 10.13109/zptm.2006.52.3.274 17156600

[24] Clever K Schepper F Pletschko T Herschbach P Christiansen H Martini J. Psychometric properties of the Fear of Progression Questionnaire for parents of children with cancer (FoP-Q-SF/PR). J Psychosomat Res. (2018) 107:7–13. 10.1016/j.jpsychores.2018.01.008 29502766

[25] Zeng ZH Wang YH Yuan RZ Chen JL Wang RD Tang J. The reliability and validity of the Chinese version of Fear of Progression Questionnaire-Short Form among patients with type 2 diabetes mellitus. J Qilu Nurs. (2022) 28:48–52. 10.3969/j.issn.1006-7256.2022.09.014

[26] Indelicato L Dauriz M Santi L Bonora F Negri C Cacciatori V. Psychological distress, self-efficacy and glycemic control in type 2 diabetes. Nutr Metab Cardiovasc Dis. (2017) 27:300–6. 10.1016/j.numecd.2017.01.006 28274728

[27] Dinkel A Herschbach P. Fear of progression in cancer patients and survivors. Recent results in cancer research. Psychooncology. (2018) 210:13–33. 10.1007/978-3-319-64310-6_2 28924677

[28] Goebel S Mehdorn HM. Fear of disease progression in adult ambulatory patients with brain cancer: prevalence and clinical correlates. Support Care Cancer. (2019) 27:3521–9. 10.1007/s00520-019-04665-9 30684045

[29] Hanprasertpong J Geater A Jiamset I Padungkul L Hirunkajonpan P Songhong N. Fear of cancer recurrence and its predictors among cervical cancer survivors. J Gynecol Oncol. (2017) 28:e72. 10.3802/jgo.2017.28.e72 28758378 PMC5641523

[30] Attridge M Creamer J Ramsden M Cannings-John R Hawthorne K. Culturally appropriate health education for people in ethnic minority groups with type 2 diabetes mellitus. Cochr Database Syst Rev. (2014) 4:CD006424. 10.1002/14651858.CD006424.pub3 25188210 PMC10680058

[31] Ding X Shen Z Zhang C Qi L Jiao Y Mao D. Impact of health educators' intervention on non-communicable diseases-related knowledge, attitude and behavior among rural residents. Zhonghua Yu Fang Yi Xue Za Zhi. (2015) 49:1098–103. 26887306

[32] Dizdar OS Gul OO Baspinar O Cander S Sisman P Eker B. Assessment of factors related to the understanding of education and knowledge of self-care among patients with diabetes mellitus: a cross-sectional prospective study. Adv Therapy. (2016) 33:1565–78. 10.1007/s12325-016-0378-6 27397589

[33] Winkley K Stahl D Chamley M Stopford R Boughdady M Thomas S. Low attendance at structured education for people with newly diagnosed type 2 diabetes: general practice characteristics and individual patient factors predict uptake. Patient Educ Counsel. (2016) 99:101–7. 10.1016/j.pec.2015.08.015 26319362

[34] Dong Y Wang P Dai Z Liu K Jin Y Li A. Increased self-care activities and glycemic control rate in relation to health education via Wechat among diabetes patients: a randomized clinical trial. Medicine. (2018) 97:e13632. 10.1097/MD.0000000000013632 30558051 PMC6319995

[35] Pal K Eastwood SV Michie S Farmer AJ Barnard ML Peacock R. Computer-based diabetes self-management interventions for adults with type 2 diabetes mellitus. Cochr Database Syst Rev. (2013) 3:CD008776. 10.1002/14651858.CD008776.pub2 23543567 PMC6486319

[36] Wild D von Maltzahn R Brohan E Christensen T Clauson P Gonder-Frederick L. A critical review of the literature on fear of hypoglycemia in diabetes: implications for diabetes management and patient education. Patient Educ Couns. (2007) 68:10–5. 10.1016/j.pec.2007.05.003 17582726

[37] Kornelius E Yang YS Lo SC Peng CH Lai YR Chiou JY. Progress of diabetes severity associated with severe hypoglycemia in Taiwan. Am J Manag Care. (2018) 24:e99–106. 29668212

[38] Torimoto K Okada Y Hajime M Tanaka K Tanaka Y. Risk factors of hypoglycemia in patients with type 2 diabetes mellitus: a study based on continuous glucose monitoring. Diabetes Technol Ther. (2018) 20:603–12. 10.1089/dia.2018.0017 30070933

[39] Kouwenhoven-Pasmooij TA Burdorf A Roos-Hesselink JW Hunink MG Robroek SJ. Cardiovascular disease, diabetes and early exit from paid employment in Europe; the impact of work-related factors. Int J Cardiol. (2016) 215:332–7. 10.1016/j.ijcard.2016.04.090 27128556

[40] Vickberg SM. The Concerns About Recurrence Scale (CARS): a systematic measure of women's fears about the possibility of breast cancer recurrence. Ann Behav Med. (2003) 25:16–24. 10.1207/S15324796ABM2501_03 12581932

[41] Seuring T Goryakin Y Suhrcke M. The impact of diabetes on employment in Mexico. Econ Hum Biol. (2015) 18:85–100. 10.1016/j.ehb.2015.04.002 25985080

[42] Hesketh T Lu L Xing ZW. The effect of China's one-child family policy after 25 years. N Engl J Med. (2005) 353:1171–6. 10.1056/NEJMhpr051833 16162890

